# Microenvironmental Regulation of Chondrocyte Plasticity in Endochondral Repair—A New Frontier for Developmental Engineering

**DOI:** 10.3389/fbioe.2018.00058

**Published:** 2018-05-15

**Authors:** Sarah A. Wong, Kevin O. Rivera, Theodore Miclau, Eben Alsberg, Ralph S. Marcucio, Chelsea S. Bahney

**Affiliations:** ^1^Department of Orthopaedic Surgery, Orthopaedic Trauma Institute, University of California, San Francisco, San Francisco, CA, United States; ^2^School of Dentistry, University of California, San Francisco, San Francisco, CA, United States; ^3^Department of Orthopaedic Surgery and Biomedical Engineering, Case Western Reserve University, Cleveland, OH, United States

**Keywords:** fracture, endochondral ossification, chondrocyte fate, developmental engineering, transdifferentiation

## Abstract

The majority of fractures heal through the process of endochondral ossification, in which a cartilage intermediate forms between the fractured bone ends and is gradually replaced with bone. Recent studies have provided genetic evidence demonstrating that a significant portion of callus chondrocytes transform into osteoblasts that derive the new bone. This evidence has opened a new field of research aimed at identifying the regulatory mechanisms that govern chondrocyte transformation in the hope of developing improved fracture therapies. In this article, we review known and candidate molecular pathways that may stimulate chondrocyte-to-osteoblast transformation during endochondral fracture repair. We also examine additional extrinsic factors that may play a role in modulating chondrocyte and osteoblast fate during fracture healing such as angiogenesis and mineralization of the extracellular matrix. Taken together the mechanisms reviewed here demonstrate the promising potential of using developmental engineering to design therapeutic approaches that activate endogenous healing pathways to stimulate fracture repair.

## Introduction

Fractures heal through two pathways: endochondral ossification and intramembranous ossification (Thompson et al., 2002; Bahney et al., 2015). Both processes begin with the differentiation of local osteochondral progenitor cells found within the periosteum and endosteum (Colnot, 2009; Duchamp de Lageneste et al., 2018). During endochondral ossification, or indirect bone healing, progenitor cells primarily derived from the periosteum differentiate into chondrocytes to form a cartilage callus between the fractured bone ends (Duchamp de Lageneste et al., 2018). This cartilage is gradually replaced with bone in a process that resembles embryonic bone development and post-natal growth. Intramembranous ossification, or direct bone healing, occurs when periosteal and endosteal progenitor cells differentiate directly into osteoblasts. Fate of the osteochondral progenitor is determined by the relative stability of the fracture site, with motion stimulating endochondral ossification and rigid microenvironments promoting intramembranous ossification (Thompson et al., 2002). In most cases, both healing pathways occur simultaneously such that a robust cartilage callus forms at the center of the fracture where the degree of motion is greatest, and intramembranous bone forms along the periosteal and endosteal surfaces (Thompson et al., 2002). Endochondral ossification is the predominant mechanism by which the majority of fractures heal and is the focus of this review (Silkstone et al., 2008; Bahney et al., 2015).

Formation of the cartilage callus functionally serves to stabilize the gap between the bone ends. To form the cartilage callus periosteal osteochondral progenitor cells migrate from the periosteum and undergo chondrogenic differentiation (Colnot, 2009). This occurs on top of the provisional fibrin matrix formed by the hematoma (Xing et al., 2010a). Growth factors produced by the hematoma promote cell migration and differentiation and also create a unique microenvironment with low pH and high lactate concentration (Wray, 1964). Formation of the hematoma and a strong pro-inflammatory response are essential to establishing a robust healing response (Park et al., 2002).

Following the initial hematoma, the subsequent steps of chondrogenesis and chondrocyte hypertrophy appear to parallel the molecular pathways involved in endochondral ossification in the growth plate during bone development (Kronenberg, 2003; Long and Ornitz, 2013). Chondrogenic programming is initiated by the expression of transcription factor Sox9, which is required for chondrogenesis (Bi et al., 1999; Akiyama et al., 2002). Sox9 regulates the expression of several chondrocyte-specific matrix components including collagen type II and aggrecan, the two predominant proteins within the cartilage matrix (Bell et al., 1997; Sekiya et al., 2000). This initial extracellular matrix is avascular and aneural until blood vessels and nerves penetrate the soft callus during later stages of healing (Gerber et al., 1999; Tatsuyama et al., 2000; Grässel, 2014; Hu et al., 2017). As chondrocytes mature, they produce collagen type X, mineralize their surrounding matrix, and undergo hypertrophy, increasing in volume and dry mass by ~20-fold (Cooper et al., 2013).

There has been a centuries-long debate regarding the subsequent fate of hypertrophic chondrocytes during endochondral bone development and repair. In the early 1800's, cartilage was believed to turn into bone (Beresford, 1981; Hall, 2015). However, in the mid-1800's, Muller and Sharpy changed this paradigm by claiming that chondrocytes are terminally-differentiated and ultimately undergo cell death, resulting in the replacement of cartilage with bone derived from a separate population of cells (Beresford, 1981; Hall, 2015). The latter model of chondrocyte fate, for the most part, dominated in textbooks and became the *de facto* model of endochondral ossification. In recent years, modern murine genetics has enabled lineage tracing studies that can more accurately follow the fate of cells. Using a combination of over five different genetic models, evidence now demonstrates that a significant portion of chondrocytes survive, proliferate, and transform into osteoblasts that derive the new bone (Bahney et al., 2014; Yang et al., 2014; Zhou et al., 2014; Jing et al., 2015; Park et al., 2015; Houben et al., 2016; Hu et al., 2017).

Pathways that regulate chondrocyte to bone conversion have practical implications on fracture healing. Importantly, since conversion of cartilage to bone is necessary for bone regeneration, it is critical to understand the molecular mechanisms regulating this process. Not only will these mechanistic data improve our understanding of impaired healing, especially in the context of hypertrophic non-unions where cartilage fails to convert to bone, but they will also enable new opportunities for therapeutic intervention through modulation of cartilage to bone transformation. Here, known and candidate molecular regulators of chondrocyte-to-osteoblast transformation, along with potential sources for these biological signals, are reviewed. Finally, we propose how tissue engineering can be used to translate the evidence reviewed here into new and improved fracture therapies.

## Fracture healing standard of care

### Bone grafting

Surgical intervention is currently the only effective treatment option for recalcitrant fractures (Bahney et al., 2015). Standard of care is to use bone autograft or allograft to stimulate healing (Hubble, 2002). Together this makes bone the second-most commonly transplanted tissue behind blood. While bone autografts stimulate strong bone repair, they come with the cost of significant donor site morbidity and limited supply. On the other hand, while bone allografts are readily available, they have significantly reduced bioactivity resulting in clinical failure associated with poor osteointegration and osteonecrosis of the graft (Brigman et al., 2004). Consequently, there is an unmet clinical need to develop pharmacologic agents, or “biologics,” which can be used either as a non-invasive alternative or in conjunction with surgical treatment to stimulate endogenous healing mechanisms and improve fracture outcomes.

### Bone morphogenetic proteins

**Figure 1:**
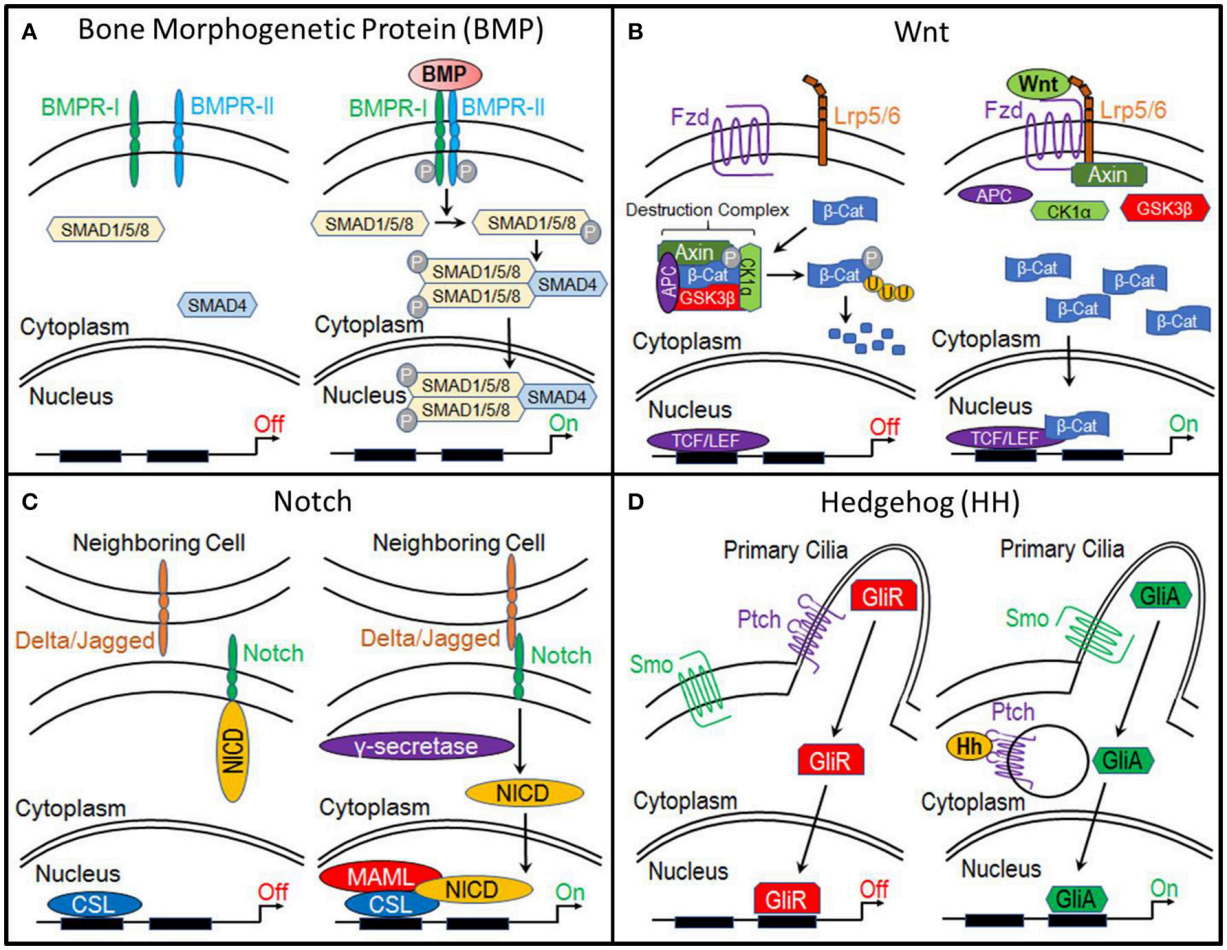
Molecular pathways. **(A)** Bone Morphogenetic Protein (BMP), **(B)** Canonical Wnt, **(C)** Notch, and **(D)** Hedgehog.

Bone morphogenetic proteins (BMPs) are currently the most common clinically-used biologics. BMP signal transduction occurs through the binding of BMP ligands to type I and type II serine/threonine kinase receptors (BMPR-I, BMPR-II). This induces phosphorylation of BMP receptors and subsequent phosphorylation of receptor SMADS (R-SMADs) 1, 5, and 8. R-SMADS then form a complex with SMAD4, enabling it to enter the nucleus where it regulates gene expression (Lin and Hankenson, 2011; Long and Ornitz, 2013; Katagiri and Watabe, 2016; Salazar et al., 2016) (Figure 1A).

Pre-clinical studies indicated that the BMP pathway was an excellent target for therapeutic development due to its role in regulating osteoblastogenesis and the ability of several BMPs to strongly induce bone formation (Hoffmann and Gross, 2001; Karsenty and Wagner, 2002; Einhorn, 2010). This led to a series of clinical trials and FDA approval of two recombinant BMPs. Recombinant human BMP2 (INFUSE®) obtained pre-market approval for use in lumbar spinal fusion and for the treatment of compound tibial fractures (Einhorn, 2010; Chrastil et al., 2013). Recombinant human BMP7, also known as Osteogenic Protein 1 (OP-1), received a Humanitarian Device Exemption for the treatment of recalcitrant long bone non-unions and for revisions of lumbar spinal fusions (Einhorn, 2010; Chrastil et al., 2013). However, although rhBMP2 has exhibited clinical success in spinal fusion, both rhBMP2 and rhOP-1 have shown less impressive results in the treatment of fracture non-unions (Einhorn, 2010). rhOP-1 has now been taken off the market and use of rhBMP2 has been significantly diminished as a result of reports of serious side effects, including heterotopic ossification and tumorigenesis, and by the expense of treatment ($5,000–$15,000 per treatment) (Einhorn, 2010; DeVine et al., 2012; Chrastil et al., 2013; Almubarak et al., 2016).

It has been postulated that the lack of clinical success with BMPs is due to limited understanding of the molecular signals responsible for regulating fracture repair and that a combination of biologics applied during the appropriate phases of the repair process will be required to effectively stimulate healing (Simmons et al., 2004; Sukul et al., 2015; Dang et al., 2016a). Furthermore, supraphysiological dosing, burse release kinetics, and rapid diffusion of BMPs are key factors contributing to heterotopic ossification (Krishnan et al., 2017). As reviewed recently, engineering scaffolds and drug delivery systems to promote sustained and local delivery of BMPs is a significant and active area of research that can translate into improved clinical outcomes (Bessa et al., 2008; Bhattacharjee et al., 2015; Agrawal and Sinha, 2017).

## Novel molecular targets for fracture healing

To study the molecular signals regulating chondrocyte-to-osteoblast transformation, we have defined the chondro-osseous border in the fracture callus as the “Transition Zone” (Hu et al., 2017). Here, mature hypertrophic chondrocytes have been shown to express classic osteogenic markers (i.e., runx2, osterix, collagen type I, osteocalcin, osteopontin) indicating that these cells adopt an osteogenic fate (Hu et al., 2017). Interestingly, a recent publication by Hu et al. demonstrated that hypertrophic chondrocytes at the Transition Zone also express pluripotency transcription factors Sox2, Oct4, and Nanog, suggesting that chondrocytes acquire a stem cell-like state during transformation (Hu et al., 2017). Sox2 was shown to play an important role during chondrocyte transformation since its deletion resulted in significantly reduced bone formation and increased cartilage retention within the fracture callus (Hu et al., 2017).

**Figure 2:**
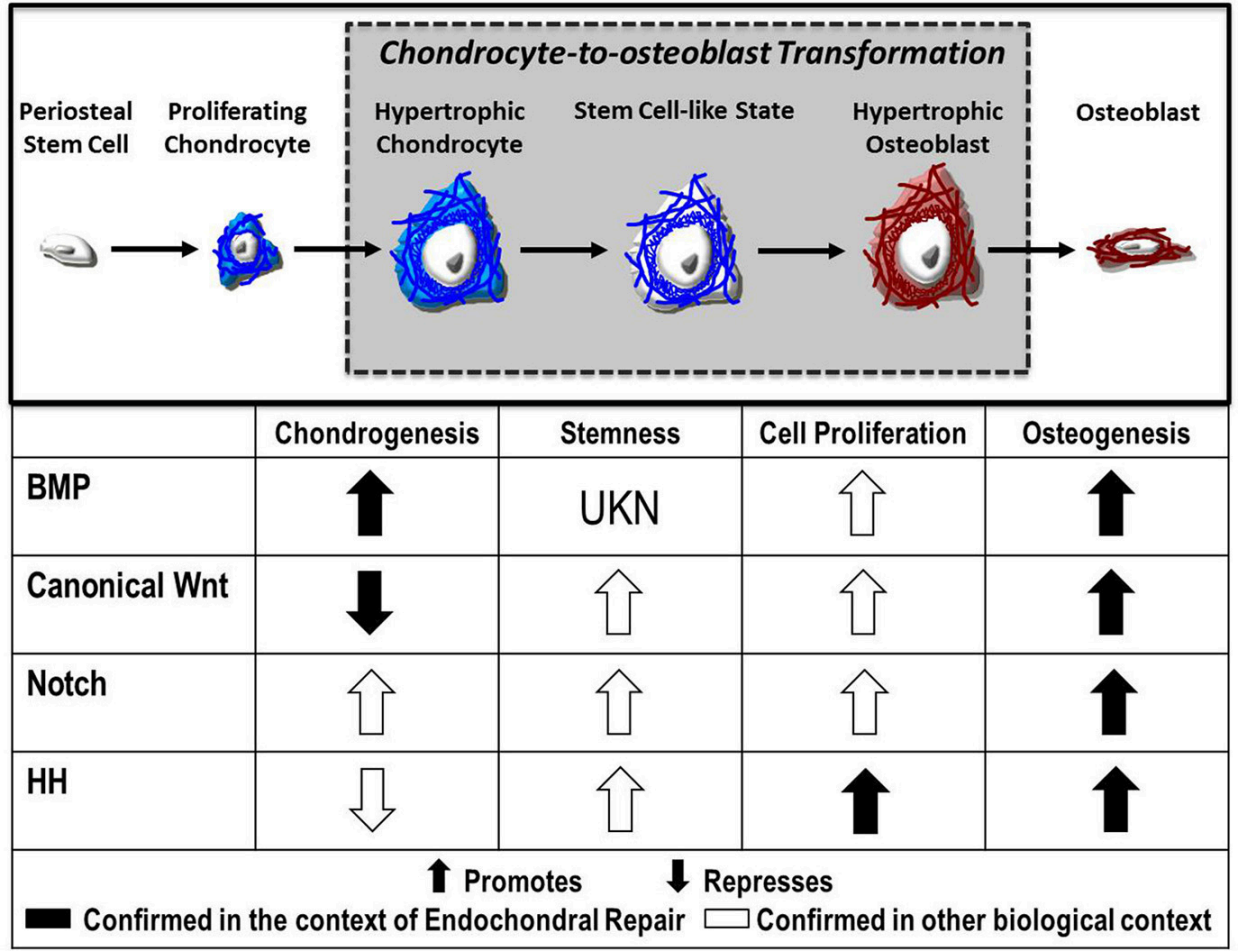
Fate of the chondrocyte. During endochondral ossification, the formation of the cartilage callus begins with the differentiation of periosteal stem cells into chondrocytes, which proliferate and mature to a hypertrophic state. These hypertrophic chondrocytes then re-enter the cell cycle, express stem cell markers, and finally transform into osteoblasts that contribute to the formation of new bone. Published evidence suggests the Bone Morphogenetic Protein (BMP), Canonical Wnt, Notch, and Hedgehog (HH) pathways as candidate regulators of chondrocyte-to-osteoblast transformation due to their effects on chondrogenesis, stemness, cell proliferation, and osteogenesis in the context of endochondral repair (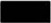) and in other biological contexts (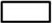).

Despite advances in our understanding of chondrocyte gene expression during transformation, the signaling mechanisms that direct this process remain largely unknown. Evidence suggests numerous molecular pathways as regulatory candidates, including canonical Wnt, Notch, FGF, and Hedgehog signaling, each of which will be explored here (Figure 2).

### Canonical wnt signaling

Wnt signaling is traditionally categorized into the β-catenin-dependent canonical pathway and the β-catenin-independent non-canonical pathways (planar cell polarity and Ca^2+^-mediated pathways), as recently reviewed (Gammons and Bienz, 2018). While some evidence suggests that the non-canonical pathways may play a role in regulating osteogenesis (Chen et al., 2007), the canonical Wnt/β-catenin pathway is the most studied and has been shown to play a dominant role in bone development and fracture repair. Thus, this review focuses on the canonical Wnt pathway.

The primary function of canonical Wnt signaling is to regulate the transcription of genes involved in cellular processes such as proliferation, differentiation, self-renewal, and survival. When this pathway is inactive, β-catenin, a transcriptional co-activator and the primary effector of this pathway, is bound by a multiprotein “destruction” complex, which consists of Axin, adenomatous polyposis coli (APC), and serine/threonine kinases glycogen synthase kinase 3β (GSK3β) and casein kinase 1α (CK1α). This destruction complex phosphorylates β-catenin, targeting it for ubiquitination and ultimately proteosomal degradation. However, when the pathway is activated by the binding of Wnt ligands to Frizzled and LRP5/6 receptors, the destruction complex is disrupted, enabling β-catenin to accumulate within the cytoplasm and translocate to the nucleus, where it interacts with members of the T-cell factor/lymphocyte elongation factor (TCF/LEF) family to activate transcription of target genes (Gammons and Bienz, 2018) (Figure 1B).

The canonical Wnt pathway has an established role in osteogenesis and skeletal formation by functioning as a molecular switch regulating lineage commitment between osteogenesis and chondrogenesis (Hill et al., 2005; Topol et al., 2009). During development, inhibition of canonical Wnt signaling through conditional deletion of β-catenin from limb and head mesenchyme using *Prx1*-CreERT, or conditional deletion from skeletogenic mesenchyme using *Dermo1*-Cre, inhibits bone formation and results in early osteoblast differentiation arrest (Day et al., 2005; Hill et al., 2005). Osteoblastogenesis halts at the osteochondral progenitor stage and cells differentiate into chondrocytes, resulting in the formation of ectopic cartilage (Day et al., 2005; Hill et al., 2005). Although cells express Runx2, an early marker of the osteoblast lineage, they fail to express osterix, indicating that these cells are incapable of committing to an osteogenic fate (Day et al., 2005; Hill et al., 2005). *In vitro* experiments inhibiting canonical Wnt signaling in mesenchymal progenitor cells provide similar findings (Hill et al., 2005).

Canonical Wnt signaling also plays a key role in directing osteogenesis during intramembranous repair (Kim et al., 2007). Using a transcortical defect model, which heals through intramembranous ossification, inhibition of Wnt signaling through adenoviral expression of Dkk1 prevented the differentiation of osteoprogenitor cells into osteoblasts and significantly reduced bone regeneration compared to controls (Kim et al., 2007). Conversely, activating the canonical Wnt pathway through deletion of pathway inhibitors (sclerostin or Axin2) significantly improved intramembranous bone formation (McGee-Lawrence et al., 2013). Furthermore, treatment of bone grafts with Wnt3a protein restored the osteogenic potential of aged bone grafts and promoted intramembranous healing of critical-sized defects in mouse calvaria and rabbit ulna (Leucht et al., 2013).

Less work has been done to determine the role of canonical Wnt signaling during endochondral bone formation and repair since traditionally the Wnt pathway is thought to promote direct osteogenesis. However, the mounting data demonstrating chondrocytes can directly form bone in development and repair (Bahney et al., 2014; Yang et al., 2014; Zhou et al., 2014; Jing et al., 2015; Park et al., 2015; Houben et al., 2016; Hu et al., 2017) suggests that canonical Wnt signaling may have a functional role in chondrocyte-to-osteoblast transdifferentiation. This was directly tested recently by Houben et al. who showed conditional deletion of β-catenin in *col10a1*-expressing hypertrophic chondrocytes resulted in significantly reduced bone, whereas stabilized β-catenin produced osteopetrotic tissue during endochondral development (Houben et al., 2016).

Since fracture repair in many ways recapitulates bone development, canonical Wnt signaling may play a similar role in regulating chondrocyte-to-osteoblast transformation during endochondral repair. Indeed, during endochondral healing, nuclear localization of β-catenin was seen in hypertrophic chondrocytes at the fracture callus Transition Zone, indicating that these cells undergo active canonical Wnt signaling (Hu et al., 2017). RT-qPCR analysis of fracture calli revealed that numerous Wnt ligands, receptors, and transduction machinery are expressed during fracture repair (Chen et al., 2007; Leucht et al., 2008). Huang et al. demonstrated that inhibition of Wnt/β-catenin signaling in chondrocytes, using an 82-amino-acid peptide called Inhibitor of β-catenin/TCF (ICAT) driven by *col2a1* expression, delayed cartilage formation and reduced bone formation (Huang et al., 2012b). Similarly, activation of canonical Wnt signaling through treatment with lithium chloride enhanced bone formation (Chen et al., 2007). Interestingly, enhanced bone regeneration was only observed when the Wnt pathway was activated at later time points, which corresponds biologically with chondrocyte-to-osteoblast transformation (Chen et al., 2007). Together, these data suggest that canonical Wnt signaling may play a role in regulating chondrocyte-to-osteoblast transformation during fracture healing.

The evidence outlined above are derived primarily from pre-clinical studies and *in vitro* systems. However, it is likely that the canonical Wnt pathway plays a similarly critical role in humans. Numerous human bone diseases are associated with mutations to components of the canonical Wnt pathway (Regard et al., 2012). Predisposition to osteoporosis has been associated with genomic polymorphisms in or close to Wnt/β-catenin signaling components (Regard et al., 2012). Loss-of-function mutations in the Wnt receptor LRP5 are associated with osteoporosis pseudoglioma (OPPG) syndrome and juvenile osteoporosis and gain-of-function mutations in the same receptor result in the opposite phenotype of high bone mass and enhanced bone strength (Einhorn, 2010; Regard et al., 2012). Sclerosteosis is a bone disease characterized by an overgrowth of bone and is caused by mutations in the gene and enhancer regions of the Wnt/β-catenin antagonist *sclerostin* (*SOST*) (Einhorn, 2010; Regard et al., 2012). Furthermore, the canonical Wnt pathway has been implicated in the context of human fracture repair since β-catenin and sclerostin levels have been shown to increase (Chen et al., 2007; Sarahrudi et al., 2012).

The canonical Wnt pathway is primed for translation. Numerous Wnt pathway regulators are being developed and several are already in clinical trials. The majority of these pathway modulators serve to activate the canonical Wnt pathway by neutralizing pathway inhibitors such as Dkk1 and sclerostin (Canalis, 2013). This indirect approach to pathway activation has been adopted primarily because direct pathway activation through treatment with Wnt ligands is clinically-irrelevant. Endogenous Wnts are hydrophobic due to palmitoylation, a form of lipidation required for the intracellular trafficking and full activation of Wnts (Willert et al., 2003; Takada et al., 2006; Janda et al., 2012). This makes Wnts challenging to extract and purify, requires that they be delivered using special liposome-based systems, and significantly increases the cost of treatment (Morrell et al., 2008). Fortunately, several of the Wnt pathway modulators acting to neutralize pathway inhibitors have shown promising osteogenic effects during clinical trials.

Of the Wnt pathway regulators currently in development, Romosozumab is closest to attaining FDA approval and is currently in Phase III clinical trials for treating osteoporosis (Regard et al., 2012; Canalis, 2013). It is a humanized monoclonal antibody that binds to and neutralizes the Wnt inhibitor sclerostin (Canalis, 2013). Studies show that treatment with Romosozumab significantly increases bone mineral density and reduces incidence of osteoporotic fractures (Canalis, 2013). Wnt pathway regulators, such as Romosozumab, could readily be repurposed for the context of fracture repair. However, the optimal dosage, timing, and the method of treatment still need to be determined.

### Notch

Like, the canonical Wnt pathway, the functional roles of Notch signaling suggest it as a candidate regulator of chondrocyte-to-osteoblast transformation. Activation of this pathway begins when the Notch transmembrane receptor binds to membrane-bound ligands (Delta or Jagged) on the surface of neighboring cells. This triggers the proteolytic cleavage of the Notch intracellular domain (NICD) by y-secretase. NICD then translocates to the nucleus where it forms a complex with and activates the transcription factor CSL, which recruits its co-activator Mastermind-like (MAML) and initiates transcription of target genes (Lin and Hankenson, 2011) (Figure 1C).

Notch signaling has been shown to promote osteoblastogenesis. *In vitro* inhibition of Notch signaling in mouse MSCs impaired osteoblast differentiation as assessed by alizarin red staining for matrix mineralization (Dishowitz et al., 2013). *In vivo*, gain-of-function Notch signaling in osteoblasts through the overexpression of NICD resulted in abnormally dense or osteosclerotic bone attributed to increased cell proliferation of immature osteoblasts (Engin et al., 2008). Similarly, loss-of-function Notch signaling in osteoblasts, through mutations to y-secretase, led to late-onset osteoporosis (Engin et al., 2008).

Notch signaling also appears to play a role in promoting hypertrophic maturation of chondrocytes. During development, inhibition of Notch signaling in chondrocytes impaired terminal stages of endochondral ossification in the limb cartilage, resulting in shorter limbs with an increased hypertrophic zone and reduced bone (Hosaka et al., 2013). In the context of disease, Notch signaling may promote osteoarthritis (OA), which resembles pathological activation of endochondral ossification (Hosaka et al., 2013). Nuclear localization of the intracellular domains of Notch-1 and -2 was observed in chondrocytes in mouse and human OA articular cartilage, indicating active Notch signaling in these cells (Hosaka et al., 2013). Functionally, inhibition of Notch signaling in chondrocytes conferred resistance to OA development in the knee joint (Hosaka et al., 2013).

Notch signaling has also been shown to play an important role during fracture repair. Notch signaling is upregulated during both intramembranous and endochondral ossification, but data suggest it is more highly activated during endochondral ossification (Dishowitz et al., 2012). During endochondral ossification, Notch signaling decreases as progenitors differentiate into chondrocytes and as chondrocytes mature to hypertrophy. However, mature hypertrophic chondrocytes at the Transition Zone re-expressed Jag1 and NICD2, indicating that these cells have re-activated the Notch pathway (Dishowitz et al., 2012). Whether the Notch pathway plays a functional role in regulating chondrocyte-to-osteoblast transformation is unknown. However, systemic inhibition of Notch signaling using the *Mx1*-Cre;*dnMAML*^fl/−^ mouse impaired fracture healing primarily due to a prolonged inflammatory phase, decreased cartilage callus formation, and decreased osteoblast and osteoclast cell density (Dishowitz et al., 2013).

### Hedgehog signaling

The Hedgehog (Hh) pathway is essential to osteogenesis. When this pathway is inactive, cell surface receptor Patched (Ptch) prevents transmembrane protein Smoothened (Smo) from entering the primary cilia. This results in the proteolytic processing of Gli transcription factors into a repressor form (GliR). GliR then enters the nucleus and prevents Hedgehog target gene expression. Hedgehog signaling is activated by the binding of Hh ligands to Patched, thus relieving Patched-mediated suppression of Smoothened through Patched endocytosis. Smoothened enters the primary cilia where it prevents Gli transcription factors from being processed. Thus, Gli remains in its full-length, active form (GliA), which translocates to the nucleus and activates expression of Hedgehog target genes (Lin and Hankenson, 2011) (Figure 1D).

Of the three Hedgehog homologs, Sonic hedgehog (Shh) and Indian hedgehog (Ihh) have been implicated in osteoblastogenesis (Ehlen et al., 2006). Shh acts at early stages of development to direct patterning and growth (Zhu et al., 2008). Ihh is involved at later stages of endochondral ossification during limb development and consequently has been studied in greater depth in the context of bone formation and repair (Ehlen et al., 2006). Indian hedgehog is a central regulator of skeletogenesis and is required for osteoblastogenesis in endochondral, but not membranous bones (Kronenberg, 2003; Hill et al., 2005; Lin and Hankenson, 2011). Ihh is primarily expressed by pre- and early hypertrophic chondrocytes, where it controls proliferation and the onset of chondrocyte hypertrophy (St-Jacques et al., 1999; Long et al., 2001, 2004; Maeda et al., 2007). During development, chondrocyte expression of Ihh triggers Runx2 expression in the periosteum, thus coupling chondrocyte differentiation/maturation with osteoblastogenesis (Hill et al., 2005; Ehlen et al., 2006).

Like canonical Wnt signaling, evidence suggests that the Hedgehog pathway also serves as a molecular switch between osteogenesis and chondrogenesis. Chimeric embryos derived from Smoothened null and wild type embryonic cells exhibited abnormal bone collar formation (Long et al., 2004). Whereas, wild type cells underwent normal osteoblast differentiation, adjacent mutant cells failed to differentiate into osteoblasts and instead exhibited chondrocyte morphology, deposited cartilaginous matrix and expressed chondrocyte markers (collagen type II and X) (Long et al., 2004).

During development, Hedgehog signaling has also been shown to play an important role in trabecular bone formation. Inhibition of Hedgehog signaling through deletion of *Smoothened* in chondrocytes prevented formation of the primary spongiosa (Long et al., 2004). This loss in trabecular bone formation correlated with lost expression of the Hedgehog target gene, *Patched1*, at the chondro-osseous junction, suggesting that Hedgehog signaling promotes chondrocyte-to-osteoblast transformation (Long et al., 2004).

The Hedgehog pathway has also been implicated in regulating chondrocyte-to-osteoblast transformation during post-natal endochondral bone growth. Gli1-CreERT2 Hedgehog reporter mice demonstrated active Hedgehog signaling in hypertrophic chondrocytes and osteoprogenitors at the chondro-osseous junction of the growth plate (Haraguchi et al., 2018). Furthermore, deletion of Ihh from growth plate chondrocytes in post-natal mice resulted in continuous loss of trabecular bone with progression of age (Maeda et al., 2007).

Hedgehog signaling has been shown to promote osteogenesis during skeletal homeostasis. Systemic inhibition of Hedgehog signaling through treatment with cyclopamine decreased bone mass in adult mice (Ohba et al., 2008). In contrast, enhanced bone formation, was observed with forced activation of Hedgehog signaling in mature osteoblasts through global Patched1 haploinsufficiency or deletion (Ohba et al., 2008). Interestingly, enhanced Hedgehog activity also resulted in excessive bone resorption due to the role of Hedgehog signaling in promoting osteoclastogenesis (Mak et al., 2008).

Evidence suggests that the hedgehog pathway promotes endochondral repair as signaling is upregulated during fracture healing (Liu et al., 2017). Furthermore, Gli1 reporter mice demonstrated that cells actively signaling through the hedgehog pathway contribute to both chondrocytes and osteoblasts during fracture healing (Shi et al., 2017). Inhibition of the Hedgehog pathway through treatment with a systemic Hedgehog inhibitor GDC-0449, delayed fracture healing (Liu et al., 2017). Chondrogenesis was unaffected, suggesting that the effects were due to Hedgehog regulation of chondrocyte transformation (Liu et al., 2017). In contrast, activation of Hedgehog signaling through local administration of a Hedgehog agonist known as Smoothened Agonist (SAG) accelerated endochondral repair due to increased chondrocyte proliferation, an enlarged cartilaginous callus, and an increased number of cells expressing osteoblast markers within the bony callus (Kashiwagi et al., 2016).

## Vasculature regulation of chondrocyte-to-osteoblast transformation

The vasculature plays a critical role during fracture repair. Whereas, the normal rate of impaired healing is 10–15%, this percentage increases to 46% when fractures occur in conjunction with severe vasculature injury (Bahney et al., 2015). The role of the vasculature begins at the outset of injury during hematoma formation where it helps to create the growth factor rich fibrin blood clot upon which periosteal stem cells differentiate to chondrocytes under a low pH, high lactate microenvironment (Wray, 1964; Xing et al., 2010a). After chondrogenic differentiation, the cartilage anlage is avascular and chondrogenic maturation happens in the absence of a regulatory role from the vasculature (Gerber et al., 1999; Tatsuyama et al., 2000; Hu et al., 2017).

In the later stages of repair, blood vessels are recruited into the cartilage fracture callus by hypertrophic chondrocytes expressing vascular endothelial growth factor (VEGF) (Gerber et al., 1999; Zelzer et al., 2002; Hu et al., 2017) and placental growth factor (PlGF) (Maes et al., 2006). Histologically, the cartilage to bone transition in the fracture callus occurs around this invading vasculature (Hu et al., 2017). Importantly, spatiotemporal expression of osteogenic genes and pluripotency transcription factors occurs in hypertrophic chondrocytes adjacent to the vasculature, suggesting that the vasculature plays a role in initiating chondrocyte-to-osteoblast transformation (Hu et al., 2017).

### Growth factor secretion

Endothelial cells from the vasculature may functionally contribute to phenotypic modulation of the chondrocyte phenotype through secretion of pro-osteogenic growth factors. For example, it has been established that vascular tissues are a direct endogenous source of BMPs (Yu et al., 2010; Matsubara et al., 2012). Functionally it has been shown that secreted factors from vascular endothelial cell conditioned media were capable of inducing matrix mineralization and up-regulating the classic osteogenic gene *osteocalcin* (Bahney et al., 2014). It is likely that BMP expression contributed to this phenotype (Bahney et al., 2014). However, more recently it was also shown that the same vascular endothelial cell conditioned media induced expression of pluripotency transcription factors (Sox2, Oct4, Nanog) indicating that an additional factor may have a role in activating a stem-like state (Hu et al., 2017). While the complete secretome of vascular endothelial cells during fracture healing has not been detailed, it is known that this secretome is site specific (Nolan et al., 2013; Rafii et al., 2016). It is possible that fracture callus endothelial cells secrete factors other than BMP that may play a role in directing osteogenesis or chondrocyte plasticity.

### Delivery of macrophages

The vasculature is also responsible for delivering inflammatory cells to the fracture callus. These include circulatory macrophages, which are recruited by pro-inflammatory cytokines [Tumor necrosis factor (TNFα), Interleukin-1β (IL-1β), and IL-6] that activate a pro-inflammatory (M1) macrophage state (Wray, 1964). This pro-inflammatory phase has been shown to improve fracture repair by promoting cell proliferation and stem cell differentiation (Xing et al., 2010b; Wang et al., 2013).

While this inflammatory response is necessary for proper healing, it must be resolved in order for healing to progress (Wang et al., 2013). A prolonged pro-inflammatory state can delay fracture repair and is an underlying factor in impaired healing in elderly animals (Lu et al., 2008; Xing et al., 2010a,b; Abou-Khalil et al., 2014; Baht et al., 2015). Resolution of the pro-inflammatory state occurs when anti-inflammatory cytokines and growth factors [IL-10, arginase, TGFβ, EGF, PDGF, VEGF] push M1 macrophages toward the M2 phenotype (Laskin, 2009). Thus, it is possible that macrophages and their inflammatory resolution may help regulate chondrocyte-to-osteoblast transformation.

## Matrix mechanobiology

Recent studies have demonstrated that the extracellular matrix (ECM) plays an active role in regulating chondrogenic and osteogenic cell fate decisions. Changes in cell fate elicit changes to the surrounding matrix, thus producing a cycle of bi-directional interactions between cells and their surrounding matrix, a phenomenon known as “dynamic reciprocity” (Bissell et al., 1982). This cross-talk is modulated by the structural, mechanical, and biochemical cues provided by the ECM.

Remodeling of the ECM during endochondral ossification is a dynamic process that transforms the cartilaginous matrix into bone. This change in ECM contributes to the phenotypic adaptation that occurs during chondrocyte-to-osteoblast transformation. The major constituents of the cartilage ECM are collagens, hyaluronan, proteoglycans, and glycoproteins (Gentili and Cancedda, 2009). Collagens account for two-thirds of the tissue's dry weight, the most abundant of which is collagen type II (Eyre et al., 2006). Collagen type II is a fibril-forming collagen that creates nonparallel crosslinks with collagens type IX and XI. These crosslinks create a robust meshwork that gives cartilage its tensile strength. Cartilage is further characterized by its high aggrecan content (Martel-Pelletier et al., 2008). Aggrecan is anchored to hyaluronan within the matrix and is a negatively charged proteoglycan that attracts water (Roughley and Mort, 2014). This attraction of water to aggrecan creates osmotic pressure within the tissue, making cartilage shock-absorbent and resistant to high-load compression (Maldonado and Nam, 2013). Together, the collagen II and aggrecan ultrastructure allows for limited but necessary deformation under compressive forces that contributes to distribution of nutrients across the avascular tissue (Muir, 1995).

**Figure 3:**
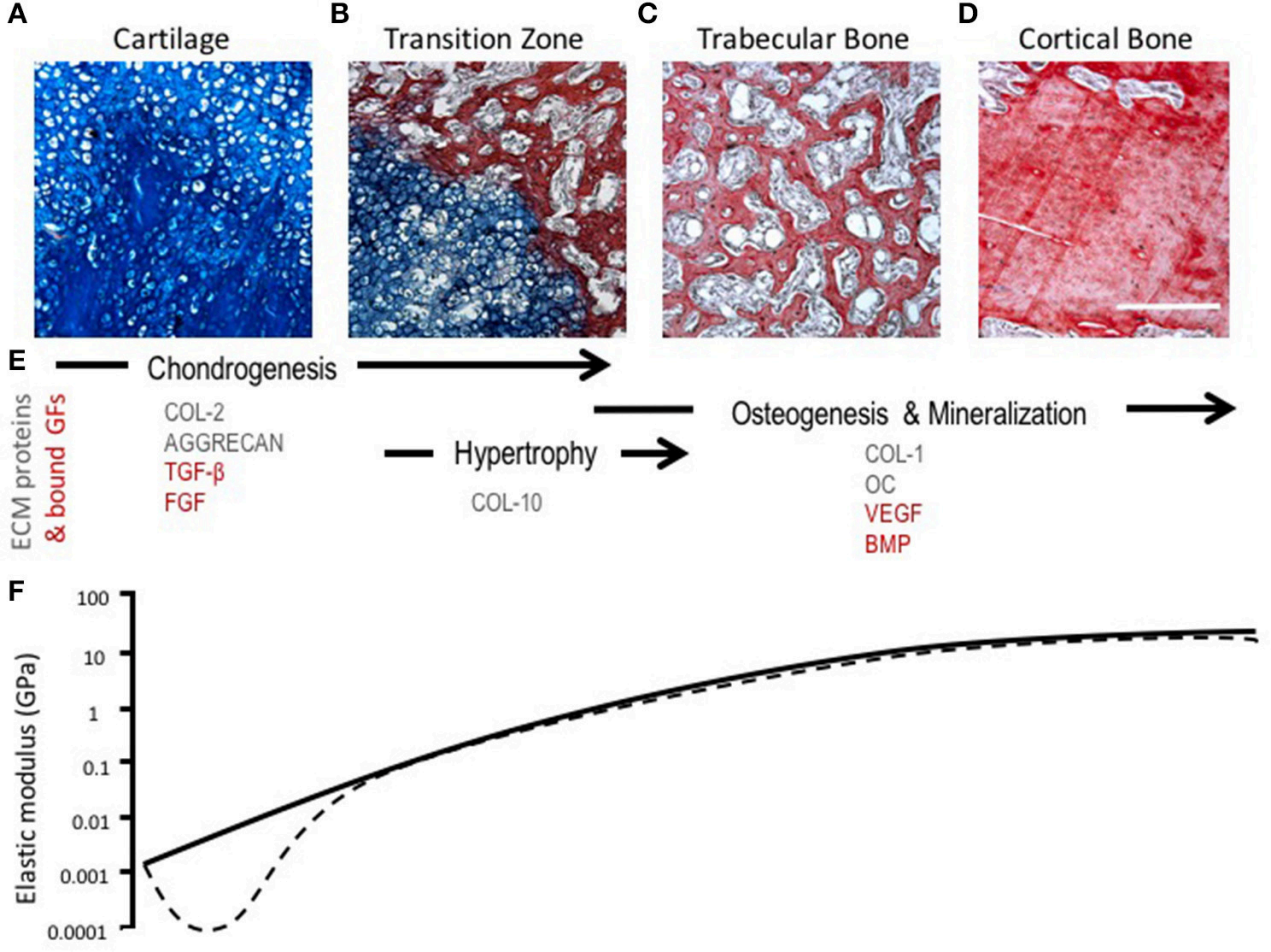
Morphological, compositional, and mechanical changes during endochondral ossification (EO). **(A–D)** HBQ histology (blue = cartilage, red = bone) of representative tissues from a murine fracture callus throughout stages of healing: **(A)** cartilage, **(B)** transition zone, **(C)** trabecular bone, and **(D)** cortical bone. Scale bar = 200 μm. **(E)** Tissue matrix components and matrix-bound growth factors corresponding to the location and phases of EO (Chintala et al., 1994; Shen, 2005; Eyre et al., 2006; Martel-Pelletier et al., 2008; Maldonado and Nam, 2013; Martino et al., 2013; Tampieri and Sprio, 2016; Tomlinson et al., 2016). **(F)** Log scale difference in elastic modulus of human samples corresponding to each tissue matrix listed above. Solid line represents normal ossification, dotted line accounts for the initial decline in elastic modulus (osteoarthritis model) (Ashman and Jae Young Rho, 1988; Rho et al., 1993; Mente and Lewis, 1994; Silver et al., 2002; Pal, 2014).

During endochondral ossification, there is a change in the amount and type of collagens present in the ECM. Chondrocyte hypertrophy is marked by the deposition of collagen type X and the up-regulation of matrix metalloproteinase-13 (MMP-13), which leads to the degradation of collagen II and aggrecan (Ortega et al., 2004; Maldonado and Nam, 2013). The loss of collagen II and aggrecan leads to a temporary reduction in tensile strength and stiffness of the tissue, which changes the mechanical microenvironment of chondrocytes and exposes the cells to greater strains that may induce phenotypic changes (Figure 3) (Stockwell, 1981; Ashman and Jae Young Rho, 1988; Rho et al., 1993; Chintala et al., 1994; Mente and Lewis, 1994; Liu et al., 2016). Proteolysis of collagen II likely contributes to chondrocyte hypertrophy and increased hydration experienced by the cartilage matrix as a consequence of a weakened fibril network losing the ability to resist the influx of proteoglycan-attracted water (Dejica et al., 2012; Akkiraju and Nohe, 2015). These changes in hydrostatic pressure could enhance mineralization of cartilage through the diffusion of ions (Tanck et al., 1999).

Numerous studies have demonstrated that chondrogenic and osteogenic gene expression can be directly modulated by compressive loading and microenvironmental stiffness, as recently reviewed (Park et al., 2011; Lv et al., 2015; Carrion et al., 2016). For example, MSCs subjected to cyclic equibiaxial strain up-reguated expression of markers specific to osteoblast differentiation and mineralization of the ECM (Thomas and el Haj, 1996; Simmons et al., 2003; Liu et al., 2016). Remarkably, when MSCs were subjected to both axial compression and sheer stress, these led to an increase in chondrogenic gene expression and elicited production and accumulation of collagen II and proteoglycan (Schätti et al., 2011; Huang et al., 2012a). Hadden et al. used adipose-derived stem cells (ASCs) cultured on hydrogels with a defined stiffness gradient to demonstrate a stiffness-dependent variation in cellular morphology, migration, and differentiation (Hadden et al., 2017). Furthermore, Engler *et al* confirmed stem cell fate plasticity by culturing MSCs on matrices with varying tissue-level elasticity. After several weeks of culture, MSCs committed to the lineage dictated by matrix stiffness such that softer, stiffer, and rigid matrices proved to be neurogenic, myogenic, and osteogenic, respectively (Engler et al., 2006). However, findings by Jha et al. suggested that high affinity adhesive ligands can serve as a substitute for a rigid matrix likely by signal transduction following focal adhesion assembly (Jha et al., 2014).

In the midst of an altering microenvironment, hypertrophic chondrocytes begin to predominantly express collagen type X. In contrast to the fibril-forming properties of collagen II, collagen X is a network-forming collagen that creates “basket weave-like” structures (Tampieri and Sprio, 2016). This collagen X ultrastructure is proposed to functionally compartmentalize matrix vesicles containing mineral and newly expressed alkaline phosphatase within the hypertrophic cartilage ECM (Kwan et al., 1997). Interactions between collagen X and matrix vesicles activate the influx of Ca^2+^ into matrix vesicles thus promoting mineralization and increasing stiffness of the matrix (Shen, 2005).

Tissue architecture, or the manner in which matrix components are structured and organized at the micro- and nanoscale, has been shown to be a factor in naïve cell differentiation. Thus, structural changes could be a driving factor for chondrocyte-to-osteoblast transformation (Healy, 2004). There have been numerous observations of matrix architecture influencing stem cell fate by controlling cell engagement with surrounding matrix and neighboring cells (Guilak et al., 2009; Ahmed and ffrench-Constant, 2016). Moreover, matrix architecture can alter cell surface receptor and cytoskeletal spatial arrangement subsequently altering ligand signaling (Ekerdt et al., 2013). For example, Lu et al. have shown that collagen type II enhances chondrogenesis in ASCs by affecting cell shape and size through the β1 integrin-mediated Rho A/Rock signaling pathway (Lu et al., 2010).

Likewise, research groups have also shown that tissue topography has the ability to guide mesenchymal stem cell fate to either chondrogenic or osteoblastic phenotypes. Shong et al. demonstrated the synergistic effect of microtopography and biochemical supplements to direct MSC fate toward an osteogenic phenotype (Guilak et al., 2009; Song et al., 2015). Additionally, work by Uskoković and Desai suggests that topography may potentially be more of a dominant factor in cell/material surface interaction than the surface chemistry or stiffness (Uskoković and Desai, 2014).

### Matrix as a growth factor reservoir

The bioavailability, local concentration, and stabilization of growth factors (GFs) within the ECM of cartilage are primarily modulated via electrostatic interactions between the negatively charged sulfate groups of proteoglycans and the positively charged surfaces of signaling molecules (Tampieri and Sprio, 2016). Moreover, GFs are immobilized by binding to heparan sulfate glycosaminoglycans, for example; Chintala et al. demonstrated that fibroblast growth factor (FGF) has a high affinity to heparan sulfate in the matrix of growth plate cartilage (Chintala et al., 1994). Similarly, Martino et al. identified various GFs from the PDGF, VEGF, TGF-β, and neurotrophin families that possess heparin-binding domains (Martino et al., 2013).

As chondrocytes mature into hypertrophic chondrocytes, they secrete VEGF to stimulate angiogenesis, alkaline phosphatase to induce mineralization, and BMPs to promote osteogenesis (Bahney et al., 2014). These growth factors are retained within the matrix due to the combination of collagen X in compartmentalizing matrix components during endochondral ossification and through interaction with the heparin and/or sulfated proteoglycans (Shen, 2005). Thus, the dynamic promiscuity of the ECM in hypertrophic cartilage likely has a role in cellular signaling affecting physiological functions of endochondral ossification.

For these reasons tissue engineers in recent years have begun to fabricate scaffolds and microparticles that are believed to mimic the release kinetics of GFs found in the cartilage ECM during endochondral ossification. Jeon et al. harnessed the high affinity GFs have to heparin by incorporating heparin into photocrosslinkable alginate gels, recapitulating matrix-growth factor interactions allowing for controlled and sustained release of therapeutic proteins (Jeon et al., 2011). Exploiting the well-documented affinity of proteins to hydroxyapatite (HAp), Dang et al. have fabricated HAp-based microparticles that exhibit sustained delivery of BMP alone as well as controlled dual delivery of BMP with TGF-β to enhance bone tissue engineering via endochondral ossification (Bernardi et al., 1972; Dang et al., 2016a,b). Likewise, glucosamine has also been incorporated into engineered scaffolds because of its effects on chondrocyte proliferation, matrix synthesis, and gene expression via modulation of TGF-β expression levels (Varghese et al., 2007; Murab et al., 2015).

As permeability is typically very low in cartilage, this further accentuates the ECM's role in acting as a reservoir for latent growth factors (Pei et al., 2011). However, in the context of OA, a degenerative joint disease that exhibits endochondral ossification signaling, cartilage ECM degradation alters TGF-β signaling due to the displacement of TGF-β by fluid influx (Blaney Davidson et al., 2007). In native cartilaginous tissue, studies have shown that the loss of latent TGF-β induces chondrocyte hypertrophy and osteogenesis (Wu et al., 2016). Similarly, MSCs seeded onto tissue-engineered cartilage undergo hypertrophic differentiation in the presence of TGF-β, while in the absence of TGF-β MSCs undergo articular cartilage differentiation (Chawla et al., 2017). To that end, we can presume that changes in the properties of the matrix, whether directly or indirectly, have a significant role in the transformation of cartilage to bone during endochondral ossification.

## Developmental engineering to recapitulate endochondral ossification

Bone injuries are extremely common with ~15 million fracture cases and over 2 million bone grafting procedures per year (Yelin et al., 2016). The current clinical gold standard for stimulating bone regeneration is to promote intramembranous bone formation through application of bone grafts, increased biomechanical stability of the fracture with additional orthopedic hardware, or less commonly, through implantation of BMP2-soaked scaffolds (INFUSE®). Given the clinical downsides of each, there is an unmet clinical need for regenerative techniques that could improve vascularized bone regeneration.

While the established clinical approaches to bone regeneration promote intramembranous bone formation, bones both develop and heal through the process of endochondral ossification during which the cartilage callus creates an angiogenic and osteoconductive scaffold for bone formation. Recent pre-clinical studies have capitalized on this, proposing therapeutic strategies that parallel the natural healing process by utilizing engineered hypertrophic cartilage grafts to stimulate bone regeneration (Scotti et al., 2010, 2013; Farrell et al., 2011; Sheehy et al., 2013, 2014; Bahney et al., 2014; Bourgine et al., 2014; Bhattacharjee et al., 2015; Dang et al., 2017). Translating these pre-clinical studies may be one strategy to improve clinical outcomes (Nishitani and Schwarz, 2014).

Further, new mechanistic understanding of endochondral ossification could have a significant impact on the design of novel therapeutic approaches to fracture healing and bone regeneration. Since we now understand chondrocytes can be a direct precursor of osteoblasts (Yang et al., 2014; Zhou et al., 2014; Jing et al., 2015; Park et al., 2015; Hu et al., 2017) stimulating transformation of chondrocytes into osteoblasts becomes a clinically-relevant therapeutic approach. Very little work has been done to understand *how* chondrocytes become osteoblasts during endochondral ossification. If we understood the extrinsic mediators of chondrocyte to osteoblast transformation, we would not only be able to engineer an ideal treatment for hypertrophic nonunions, but we could also accelerate fracture healing under normal conditions.

## Author contributions

SW, KR, and CB drafted the primary text. CB, TM, and RM financially supported this manuscript. All authors contributed to making the figures, editing the text, and approving the manuscript.

### Conflict of interest statement

The authors declare that the research was conducted in the absence of any commercial or financial relationships that could be construed as a potential conflict of interest.
